# Maternal Vitamin D Status Correlates to Leukocyte Antigenic Responses in Breastfeeding Infants

**DOI:** 10.3390/nu14061266

**Published:** 2022-03-17

**Authors:** Danforth A. Newton, John E. Baatz, Katherine E. Chetta, Preston W. Walker, Reneé O. Washington, Judy R. Shary, Carol L. Wagner

**Affiliations:** Department of Pediatrics/Neonatology, Shawn Jenkins Children’s Hospital, Medical University of South Carolina, Charleston, SC 29425, USA; baatzje@musc.edu (J.E.B.); chetta@musc.edu (K.E.C.); walkerp@musc.edu (P.W.W.); washinro@musc.edu (R.O.W.); sharyj@musc.edu (J.R.S.); wagnercl@musc.edu (C.L.W.)

**Keywords:** vitamin D, breastfeeding, immunity, childhood health, dietary supplementation

## Abstract

It is unknown if vitamin D (vitD) sufficiency in breastfeeding mothers can lead to physiological outcomes for their children that are discernible from infant vitD sufficiency per se. In a 3-month, randomized vitD supplementation study of mothers and their exclusively breastfeeding infants, the effects of maternal vitD sufficiency were determined on infant plasma concentrations of 25-hydroxyvitamin D (i.e., vitD status) and 11 cytokines. An inverse correlation was seen between maternal vitD status and infant plasma TNF concentration (r = −0.27; *p* < 0.05). Infant whole blood was also subjected to in vitro antigenic stimulation. TNF, IFNγ, IL-4, IL-13, and TGFβ1 responses by infant leukocytes were significantly higher if mothers were vitD sufficient but were not as closely correlated to infants’ own vitD status. Conversely, IL-10 and IL-12 responses after antigenic challenge were more correlated to infant vitD status. These data are consistent with vitD-mediated changes in breast milk composition providing immunological signaling to breastfeeding infants and indicate differential physiological effects of direct-infant versus maternal vitD supplementation. Thus, consistent with many previous studies that focused on the importance of vitD sufficiency during pregnancy, maintenance of maternal sufficiency likely continues to affect the health of breastfed infants.

## 1. Introduction

In addition to its well-known roles in calcium metabolism and bone growth [1,2,3], vitD has important roles in the development and function of the infant immune system [4,5]. Infants with vitD deficiency have strong associations with respiratory infections (including respiratory syncytial virus) and chronic lung diseases such as bronchopulmonary dysplasia. VitD sufficiency, including during pregnancy, is strongly associated with lower risks of wheezing and atopic diseases such as asthma and some allergies in early childhood [6,7,8,9,10,11,12]. 

We have shown in previous studies that infants can reach vitD sufficiency through direct supplementation or through breastfeeding from vitD-sufficient mothers [13,14,15,16]. However, it is unknown if these routes of achieving sufficiency result in different physiological outcomes for the child and, thus far, discernment of these potential differences has proven elusive. 

Cholecalciferol (vitD_3_) is a steroid prohormone that can be synthesized in the skin after sunlight exposure or obtained through diet or supplementation [5]. It is also found in breast milk and, along with the additional dietary form ergocalciferol (vitD_2_), is the major vitD metabolite transferred to the infant during breastfeeding [17]. VitD is then converted into 25-hydroxyvitD (25-D, 25-hydroxy-D_3_ or -D_2_ forms), primarily by the liver. This is the major circulating metabolite, and its concentration is measured as vitD status [18]. VitD sufficiency is currently defined by the Endocrine Society as serum 25-D concentrations ≥30 ng/mL (75 nmol/L) [19]. 25-D is converted into the active metabolite 1,25-dihydroxyvitD (1,25-D) by the kidney (for circulating, endocrine function) or by many other tissues (including most immune cells) for autocrine/paracrine functions [20,21]. 1,25-D activates the vitD receptor (VDR) to regulate expression of hundreds of known genes involved in diverse physiological processes, including calcium homeostasis, immune regulation, growth/development, redox balance, metabolism, epigenetic control, cell signaling, and proliferation [3,18,22,23,24].

In numerous studies, vitD sufficiency has been shown to affect the composition of breast milk, including oligosaccharide, lipid, and protein content, immune signature (cells, regulatory and epigenomic signals), and microbiome [25,26,27,28,29]. Based on our previous vitD supplementation trials [13,14,15,16], in the ongoing pilot clinical study presented here, breastfeeding infants achieve vitD sufficiency over a 3-month period either through direct supplementation (400 IU vitD_3_/d) or by high-dose supplementation of mothers alone (6400 IU/d). The ultimate goals of this study are to determine the potential benefits of maternal vitD repletion on breast milk composition and how it may improve both innate and adaptive immune development of the infant and overall health status during early childhood. A major challenge has been to associate measurable infant study parameters and outcomes with maternal vitD status, especially given the very small quantities of infant blood usually available in such studies.

## 2. Materials and Methods

### 2.1. Clinical Study

Blood samples analyzed herein were provided by 74 mothers and their 1-month-old, exclusively breastfeeding infants enrolled in a 3-month, randomized vitD supplementation study at the Medical University of South Carolina. Mothers gave written informed consent for participation (PRO #00050609) and agreed to exclusively breastfeed for the study duration. The large majority of subjects self-identified as Hispanic or non-Hispanic Caucasian.

In blinded cohorts, breastfeeding infants and their mothers in the control group each received 400 IU vitD_3_/d (via baby or prenatal vitamins; mothers also received placebo gummy), or in the treatment group, infants received placebo, and their mothers alone received high-dose vitD supplementation of 6400 IU vitD_3_/d (prenatal vitamin plus 6000 IU vitD gummy). These doses were based on our previous vitD supplementation trials [13,14,15,16]. Breast milk, maternal and infant blood were collected at baseline (enrollment) and 3 months later. Detailed maternal dietary information and skin densitometry (as estimation of sun exposure) were obtained at each study visit for further analyses of vitD sources at study completion. Due to difficulty in enrolling subjects, factors such as seasonal exclusivity were not possible in the study, but these data will be used in final analyses at completion. Study enrollment is ongoing, and the authors are still blinded to treatment groups.

### 2.2. Sample Collection and Vitamin D Measurements

Maternal blood was collected in K_2_EDTA tubes and infant blood in sodium heparin tubes at 2 time points: visit 1 (V1), 1 month old at study enrollment; V4 (4 months old) at study completion. Portions of blood after centrifugation were collected and cryopreserved in aliquots as plasma. For some infant subjects, the majority of each blood sample was used fresh for in vitro antigenic stimulation, as described below. 

A direct radioimmunoassay developed in the Hollis laboratory and manufactured by originally manufactured by Diasorin Corp. (cat.#KIR1971, Immuno-Biological Labs, Minneapolis, MN, USA) was used to measure total maternal or infant plasma 25-D (D_3_ and D_2_), herein interchangeably termed “vitD status” [30]. Based on Endocrine Society recommendations, vitD deficiency was defined as serum 25-D < 50 nmol/L (20 ng/mL), insufficiency as ≥50 to <75 nmol/L (≥20 to <0 ng/mL), and sufficiency as ≥75 nmol/L (≥30 ng/mL) [19].

### 2.3. Plasma Cytokine Measurements

Cryopreserved aliquots of infant plasma taken at V1 or V4 were used for measurements of 11 cytokines using the an electrochemiluminescence platform (Meso Scale Discovery-MSD, Rockville, MD, USA). For 10 analytes, V-plex Proinflammatory Panel 1 human kits were used (cat# K15049D-1, MSD). Separately, U-plex human TGFβ1 kits (cat# K151XWK-1, MSD) were used for that analyte (samples here were acid-activated before assay). Plasma samples were diluted 3-fold in assay buffer before use. Protocols were performed according to the manufacturer’s instructions, and results were measured and calculated using a MESO QuickPlex SQ 120 machine (MSD). Plasma cytokines were measured in 74 infants at study enrollment and 58 infants at study completion.

### 2.4. In Vitro Antigenic Stimulation and Cytokine Measurements

For in vitro antigenic stimulation of infant whole blood, a 6× antigen stock mixture of 6 µg/mL *E. coli* lipopolysaccharide (LPS) O111:B4 (cat.# L4391, Sigma-Aldrich, St. Louis, MO, USA), 60 ng/mL phorbol 12-myristate 13-acetate (PMA) (cat.# 74042, StemCell Tech, Cambridge, MA, USA), and 6 µg/mL ionomycin (cat.# 73722, StemCell Tech) in serum-free RPMI medium (cat.# 61870036, Thermo Fisher, Waltham, MA, USA) was used. Using maternal whole blood as test samples, this formulation, based on those in previous protocols [31,32,33], was optimized to simultaneously increase secretion of all 10 cytokines in the MSD Proinflammatory Panel 1 kit. A total of 38 infant blood samples were subjected to this analysis, 18 from V1 and 20 from V4.

Briefly, 67 µL of heparinized infant whole blood (within 2 h after draw), 100 µL of RPMI medium, and 33 µL of the 6× antigen stock were combined in wells of a 96-well, flat-bottomed tissue culture plate. For controls, a blank stock mix (RPMI with corresponding amounts of ethanol and dimethyl sulfoxide used to dissolve the phorbol ester and ionomycin reagents) was used in place of the antigenic mixture. For some samples, additional wells were supplemented with 50 ng/mL 25-D_3_ (cat# 17938, Sigma Aldrich) during antigenic (or control) incubation. Plates were incubated at 37 °C in a humidified, 95% air/5% CO_2_ incubator for 20 h. Culture supernatants were then collected from each well by centrifugation (2000× *g*, 15 min) and stored at −80 °C until assayed for cytokines.

The same Meso Scale cytokine kits used for plasma measurements were used to assay supernatants after antigenic stimulation. Supernatants from vehicle controls were analyzed directly; those from stimulated cultures were diluted 10-fold. Results from these assays were normalized by appropriate dilution factors to directly compare to plasma cytokine values.

### 2.5. Statistics and Data Analyses

The primary outcome variables were infant cytokine levels in the plasma and whole blood cell culture supernatants after in vitro stimulation. Due to the blinding of vitD supplementation cohorts to the investigators in the ongoing study, the independent variable used in all analyses was total circulating 25-D concentrations in mothers and infants at the two time points of 1 month (baseline) and 4 months. For analyses of variance, *t*-tests (paired or unpaired), ANOVA, or Mann–Whitney U tests were used, as appropriate. Linear correlations and regressions were determined by calculating Pearson’s product-moment correlation coefficient (r). *p*-values of <0.05 were considered significant. Normality distributions for each data set were verified by calculating W-statistics and associated *p*-values by Shapiro–Wilk, Shapiro–Francia, or Anderson–Darling tests. When appropriate, statistically-calculated outlier values were removed using Grubbs’ test (extreme studentized deviate) or interquartile range methods (Tukey’s fences or Moore and McCabe). GraphPad software (San Diego, CA, USA) was used for statistical calculations; Discovery Workbench 4.0 software (MSD) was used for immunoassay analyses.

## 3. Results

### 3.1. Clinical Study of vitD Supplementation

Mothers and their 1-month-old, exclusively breastfeeding infants were enrolled in a randomized 3-month study designed to determine if maternal vitD sufficiency compared to infant vitD sufficiency alone is associated with measurable differences in infant immune responses. In controls, breastfeeding infants and mothers each received 400 IU vitD_3_/d; in the treatment group, infants received placebo, and their mothers alone received high-dose vitD supplementation of 6400 IU vitD_3_/d. Due to the continued blinding of these vitD supplementation cohorts to the investigators in the ongoing study, the independent variable used in all analyses herein was total circulating 25-D (25-hydroxyvitD_3_ and -hydroxyD_2_) concentrations in mothers and infants at the two time points of 1 month (V1, baseline) and 4 months (V4). Maternal and infant plasma was collected at study enrollment (74 pairs) and completion (58 pairs) to determine current vitD status (i.e., circulating total 25-vitD concentration at the time of blood draw) and infant circulating cytokine concentrations. Whole blood from 26 of the infant subjects was also used for in vitro assays to measure cytokine release after antigenic stimulation. 

**Figure 1:**
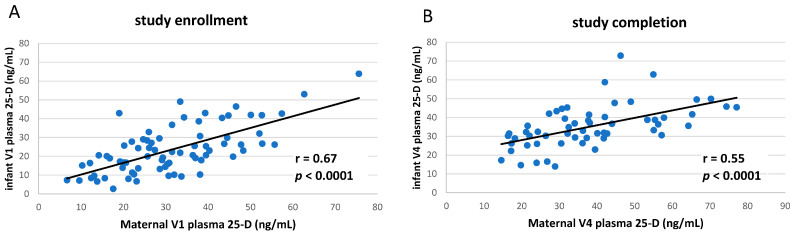
Correlation of maternal and infant 25-hydroxyvitamin D during study. Circulating concentrations of total 25-hydroxyvitamin D (vitD status) measured in plasma obtained from mothers and their breastfeeding infants at (**A**) V1 study enrollment or (**B**) V4 completion, 3 months later (all subjects; blinded study cohorts). Pearson linear correlations shown.

Maternal and infant subjects fell into three categories of vitD status at baseline or study completion [19]: deficiency (plasma 25-vitD ≤ 20 ng/mL), insufficiency (21–29 ng/mL), or sufficiency (≥30 ng/mL) (Table 1). Infant plasma 25-vitD concentrations were closely correlated to those of their mothers (*p*-value < 0.0001) at both study enrollment and completion (Figure 1A,B).

### 3.2. Circulating TNF Concentrations in Breastfeeding Infants Were Inversely Correlated to Maternal Vitamin D Status

**Figure 2:**
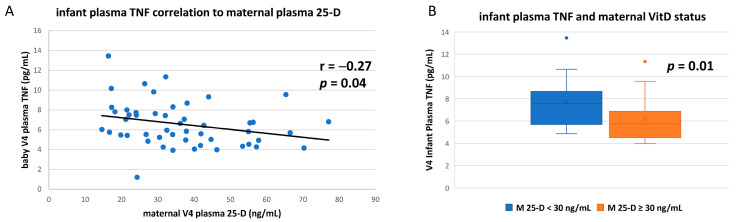
Infant plasma TNF correlation to maternal vitD status. (**A**) From blood draws at study completion, plasma concentrations of total 25-D from mothers were compared to plasma concentrations of the TNF cytokine in their breastfeeding infants (Pearson linear correlation). (**B**) Mean infant plasma TNF concentrations compared based on maternal vitD sufficiency (i.e., plasma 25-D ≥ 30 ng/mL) (unpaired *t*-test).

Using an electrochemiluminescence platform (Meso Scale Discovery, Rockville, MD, USA), circulating concentrations of 11 cytokines (interleukin (IL)-1β, IL-2, IL-4, IL-6, IL-8, IL-10, IL-12, IL-13, IFNγ, TNF, and TGFβ1) were measured in 74 infants at study enrollment and 58 infants at study completion. Regardless of infant or maternal vitD status, the concentrations of numerous measured cytokines in the growing infants changed significantly over the 3-month study period (Table 2). When concentrations of each cytokine were compared to plasma 25-D concentrations, only the proinflammatory cytokine TNF showed any significant correlation with either maternal or infant vitD status (Figure 2A). Infant plasma TNF was significantly inversely correlated to maternal vitD status (r = −0.27, *p* = 0.04), and mean infant TNF concentrations were significantly lower if their mothers were vitD sufficient (Figure 2B).

Infant plasma TNF was also correlated to the infant’s own vitD status, but not as closely as to that of the mother (r = −0.23; *p* = 0.08), and infant plasma TNF was also lower if with infant vitD sufficiency, but differences did not reach significance (*p* = 0.17) (data not shown).

### 3.3. Numerous Cytokine Responses after In Vitro Antigenic Stimulation of Breastfeeding Infant Leukocytes Were Correlated to Maternal Vitamin D Status

From a subset of 26 individual breastfeeding subjects in the vitD supplementation study, infant whole blood was stimulated in vitro for 20 h with the antigenic stock mixture of lipopolysaccharide, phorbol ester, and ionomycin (or vehicle control) to elicit immune responses. Culture supernatants were then measured for the secretion of 11 cytokines. Due to the very limiting amounts of infant blood available (usually less than 0.5 mL after plasma collection and clinical chemistry requirements), this protocol and composition of the antigen mixture were tested and optimized using maternal blood to simultaneously increase secretion of 10 cytokines (TGFβ1 was analyzed separately). A total of 38 infant blood samples were subjected to this analysis, 18 from V1 and 20 from V4 (Table 3). After 20 h in culture, no mean cytokine concentration, whether from control or antigen-stimulated blood, showed significant differences between V1 and V4 samples.

**Figure 3:**
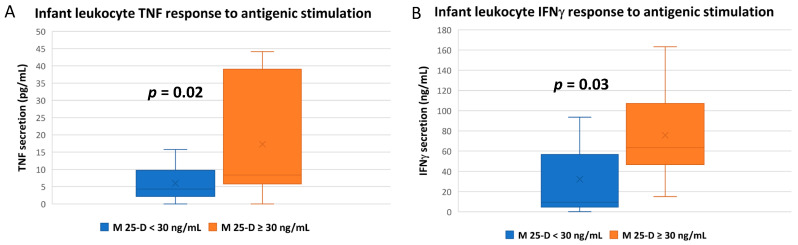
TNF and IFNγ responses of infant leukocytes to in vitro antigenic stimulation. Whole blood from breastfeeding infants was stimulated with antigen stock mixture for 20 h; culture supernatants were then collected for cytokine measurement by electrochemiluminescence. Mean cytokine concentrations compared between infants based on maternal vitD sufficiency (maternal plasma 25-D ≥ 30 ng/mL; unpaired *t*-tests) at the corresponding time of blood draw. (**A**) Mean infant TNF. (**B**) Mean infant IFNγ.

Using this in vitro antigenic stimulation method with infant whole blood, the mean TNF response of infant leukocytes was significantly higher if their mothers were vitD sufficient (Figure 3A; Tables S2 and S3), while the association with infant’s own vitD status was insignificant (*p* = 0.22; Table S2). Similarly, the infant IFNγ response to antigenic stimulation was higher with maternal vitD sufficiency (Figure 3B), but not the infant’s own vitD sufficiency (*p* = 0.29; Table S2).

**Figure 4:**
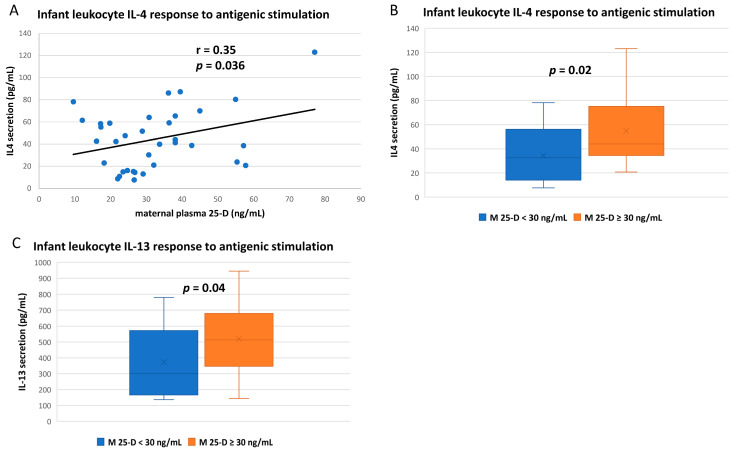
IL-4 and IL-13 responses of infant leukocytes to in vitro antigenic stimulation. Whole blood from breastfeeding infants was stimulated with an LPS-phorbol ester-ionomycin mixture for 20 h; culture supernatants were then collected for cytokine measurement by electrochemiluminescence. (**A**) Infant IL-4 release after stimulation correlated to maternal plasma 25-D (Pearson correlation). (**B**) Mean IL-4 and (**C**) IL-13 responses after antigenic stimulation compared between infants based on maternal vitD sufficiency (maternal plasma 25-D ≥ 30 ng/mL) at the corresponding time of blood draw (unpaired *t*-test).

The IL-4 and IL-13 responses of stimulated infant whole blood were also correlated to maternal vitD status (Figure 4). Direct linear correlation of IL-4 secretion to maternal plasma 25-vitD concentration reached significance (Figure 4A), but IL-4 was not correlated to the infant’s own plasma 25-vitD (*p* = 0.3, Table S1). For both IL-4 and IL-13, mean infant secretion after antigenic stimulation was higher if mothers were vitD sufficient (Figure 4B,C) but was not significantly associated with infant vitD sufficiency (*p* = 0.64 and 0.51, respectively; Table S2).

**Figure 5:**
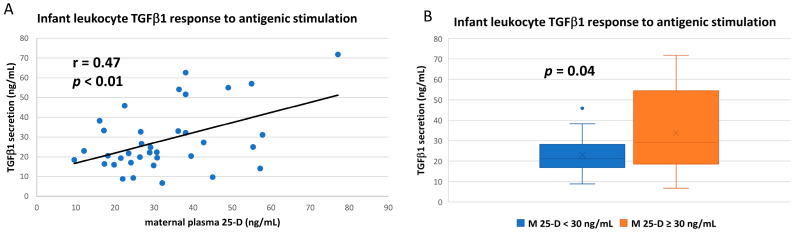
TGFβ1 responses of infant leukocytes to in vitro antigenic stimulation. Whole blood from breastfeeding infants was antigen-stimulated and culture supernatants analyzed for TGFβ1 content. (**A**) Cytokine release after stimulation correlated to maternal plasma 25-D (Pearson correlation). (**B**) Mean TGFβ1 responses after antigenic stimulation compared between infants based on maternal vitD sufficiency (maternal plasma 25-D ≥ 30 ng/mL) at the corresponding time of blood draw (unpaired *t*-test).

Though all activated leukocytes express TGFβ1, unlike the other cytokines measured herein, the majority of the circulating protein is derived from non-leukocyte sources [34] and is found at much higher plasma concentrations (Table 2). However, when measuring the in vitro leukocyte response per se after antigenic challenge, infant TGFβ1 response was directly correlated to maternal vitD status (Figure 5A; Table S1), and mean TGFβ1 secretion was significantly higher if mothers were vitD sufficient (Figure 5B; Tables S2 and S3). TGFβ1 responses were not as closely correlated to infant’s own vitD status (correlation *p* = 0.07; infant vitD sufficiency *p* = 0.08; Tables S1–S3).

### 3.4. IL-10 and IL-12 Responses after In Vitro Antigenic Stimulation of Breastfeeding Infant Leukocytes Were Correlated to Infant’s Own vitD Status

**Figure 6:**
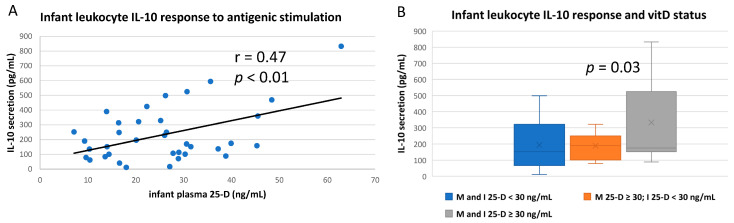
IL-10 responses of infant leukocytes to in vitro antigenic stimulation. Whole blood from breastfeeding infants was antigen-stimulated and culture supernatants analyzed for IL-10 content. (**A**) Cytokine release after stimulation correlated to infant’s own plasma 25-D (Pearson correlation). (**B**) Mean IL-10 responses after antigenic stimulation compared between groups based on both infant (I) and maternal (M) vitD sufficiency (plasma 25-D ≥ 30 ng/mL) at the corresponding time of blood draw (study enrollment or completion).

Breastfeeding infant leukocyte IL-10 secretion after antigenic challenge appeared to be more closely correlated to the infant’s own vitD status (Figure 6A) rather than to maternal status, which did not quite reach significance (*p* = 0.08; Table S1). Furthermore, by comparing different groups of dyad subjects, it seems likely that mean IL-10 secretion was significantly higher only if the infant was vitD sufficient per se (Figure 6B; there were not enough maternal insufficient–infant sufficient dyads to analyze this group). 

**Figure 7:**
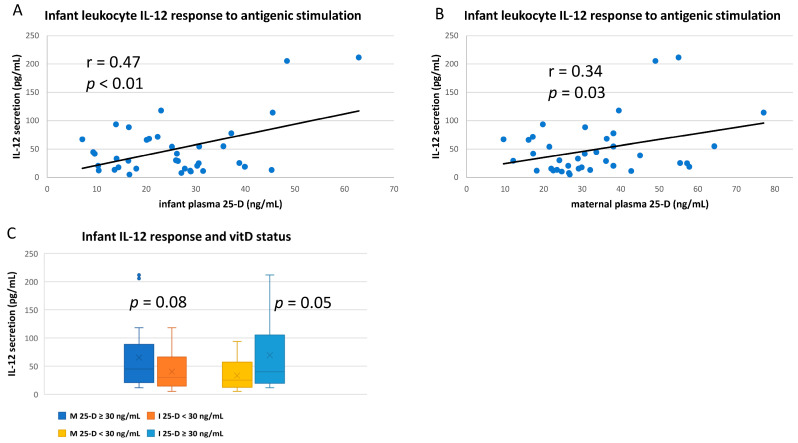
IL-12 responses of infant leukocytes to in vitro antigenic stimulation. Whole blood from breastfeeding infants was antigen-stimulated and culture supernatants analyzed for IL-12 content. Cytokine release after stimulation correlated to (**A**) infant’s own plasma 25-D or (**B**) maternal plasma 25-D. (**C**) Mean infant IL-12 responses after antigenic stimulation compared between groups based on both infant (I) and maternal (M) vitD sufficiency (plasma 25-D ≥ 30 ng/mL) at the corresponding time of blood draw.

Similarly, IL-12 response of infant’s whole blood to antigenic challenge appeared to correlate with both infant and maternal vitD status (Figure 7A,B). Likewise, vitD sufficiency of either mother or infant was associated with higher infant IL-12 response, though differences did not quite reach significance (Figure 7C).

### 3.5. Some Antigenic Responses Had No Apparent Relationship to Either Maternal or Infant vitD Status

Using the antigenic whole blood stimulation protocol, no correlations between infant or maternal vitD status reached statistical significance with respect to infant IL-1β, IL-2, IL-6, or IL-8 secretion (Tables S1 and S2).

Additionally, in a smaller group of assays (*n* = 13) in which infant whole blood cultures during antigenic stimulation were supplemented with 50 ng/mL 25-D_3_, no significant differences in cytokine measurements were seen between stimulated cultures with or without exogenous 25-D_3_ supplementation (Table S4).

## 4. Discussion

VitD sufficiency during pregnancy has been clearly shown to have numerous health benefits to both mothers and their developing children and is also associated with significant reductions in pregnancy complications [2,23,35,36,37,38,39,40,41,42,43]. Furthermore, sufficiency continues to be important for mother and infant after birth [1,6,7,9,14,20,44,45,46,47,48,49]. However, for the infant, it has yet to be definitively established if different routes to vitD sufficiency (e.g., through supplementation or breastfeeding from a vitD-sufficient mother) can lead to discriminatory differences in health.

In this pilot clinical study, exclusively breastfeeding infants achieved vitD sufficiency over a 3-month period either through direct supplementation of both infants and mothers (400 IU vitD_3_/d, likely to result in infant, but not maternal sufficiency) or by high-dose supplementation of mothers alone (6400 IU/d; likely to result in both maternal and infant sufficiency), a method we have successfully established previously [13,14,15,16,50]. The results reported herein have provided clear evidence that breast milk composition associated with maternal vitD sufficiency can result in discernible immunological differences in their breastfeeding infants in the first few months after birth that were not attributable to the infant’s own vitD sufficiency. As measured by the release of numerous well-characterized cytokines by infant leukocytes after antigenic stimulation, maternal vitD sufficiency per se, rather than the infant’s own vitD sufficiency, was associated with most enhanced leukocyte reactions. Additionally, as we have shown in previous studies, infants can reach vitD sufficiency through direct supplementation or through breastfeeding from vitD-sufficient mothers [13,14,15,16,50]. Our results here are consistent with the concept that these different routes to infant vitD sufficiency can result in discriminatory physiological effects in early childhood. 

Though immune signals of mother to baby through breast milk are also known and partially understood, especially regarding the direct transmission of antibodies, the potential roles of maternal vitD sufficiency during breastfeeding are not fully known [1,25,51]. However, in numerous studies, vitD sufficiency has been shown to affect the composition of breast milk, including oligosaccharide, lipid, and protein content; immune signature (cells, regulatory and epigenomic signals); and microbiome [25,26,27,28,29]. A major challenge has been to associate measurable infant study parameters with maternal vitD status, especially given the very small quantities of infant blood usually available to researchers.

For this study, using less than 0.5 mL of infant whole blood, we developed an assay to affect the release of numerous cytokines through in vitro antigenic stimulation [31,32,33]. This was achieved using an optimized mixture of LPS, phorbol ester, and ionomycin in overnight cell culture that would result in significant increases in the secretion of 10 cytokines. These included representative examples of cytokines traditionally associated with leukocyte immune responses termed either “innate” (e.g., those released by neutrophil or monocyte/macrophage activation) or “adaptive” (e.g., lymphocyte responses), though many of these cytokines are expressed by numerous cells and highly-affected by cell-to-cell communication [31,32,33]. Though the more common approach of using extracted peripheral blood mononuclear cells (PBMC, primarily lymphocytes and monocytes) for such studies are useful to discern individual cellular responses and allow for cryopreservation, these frequently require higher blood sample volumes and are likely less representative of physiological antigen responses, especially within the in vivo circulation. For example, PBMC do not include neutrophils, the most abundant leukocyte in circulation, and their use would not involve as many possible effects of cell-to-cell communication during the activation and course of the immune response. This whole blood assay also involves little cell manipulation and includes some autologous plasma missing from purified PBMC, further enhancing simulation of in vivo conditions. 

Among the cytokines measured in our study, a discernable association between maternal vitD status (circulating 25-hydroxyvitamin D concentration; ≥30 ng/mL considered “sufficiency”) and plasma cytokine concentrations in breastfeeding infants at study completion was seen only with tumor necrosis factor α (TNF). After 3 months in the study, an inverse correlation was seen between maternal vitD status and breastfeeding infant plasma TNF concentration (r = −0.27; *p* < 0.04). These results are consistent with the concept that plasma TNF in breastfeeding infants could be a good predictive marker for responses in vitD supplementation studies. Conversely to TNF plasma concentrations, maternal vitD sufficiency was associated with a significantly larger infant TNF response (mean ~3-fold) to in vitro antigenic stimulation compared to those with vitD insufficient mothers. 

TNF is a major proinflammatory cytokine primarily released by macrophages to activate other cells to initiate an immune response [52]. It is also a primary pyrogen at sites of inflammation, an adipokine associated with insulin resistance, and is implicated in numerous autoimmune diseases and chronic inflammation, including severe cases of COVID-19 [52,53,54]. Numerous studies in adults have associated vitD sufficiency with a reduction in TNF-associated inflammation in asthma, rheumatoid arthritis, heart disease, inflammatory bowel disease, psoriasis, and development of metabolic syndrome [48,52,55,56].

The infant IFNγ response to antigenic stimulation was also significantly higher with maternal vitD sufficiency (mean ~2.2-fold), but not an infant’s own vitD sufficiency. IFNγ is a primary indicator of lymphocyte activation and is also produced by NK cells and macrophages. It initiates and propagates both adaptive and innate responses to antigenic stimulation and has antiviral and immunoregulatory functions [57]. In adults, vitD is generally thought to partially inhibit TH1 lymphocyte activation by blocking IFNγ activation and can also synergize with the cytokine in promoting immunological tolerance [58,59].

Both IL-4 and IL-13 responses to antigenic stimulation were also significantly higher in blood from infants of vitD-sufficient mothers. These cytokines are both produced by activated TH2 lymphocytes and have many overlapping functions in B-cell activation [60]. In various adult studies, vitD has been shown to inhibit and stimulate IL-4 activation in various immune responses and has been investigated as a treatment for IL-13-associated atopic dermatitis [61,62].

Unlike the other cytokines measured in this study, the majority of plasma TGFβ1 is derived from non-leukocyte sources, though all activated leukocytes (but primarily macrophages and T-reg cells) can express TGFβ1 as well [34]. After the antigenic challenge, infant leukocyte TGFβ1 response was strongly correlated to maternal vitD status, and mean TGFβ1 secretion was significantly higher if mothers were vitD sufficient. TGFβ has multiple roles in cellular proliferation and differentiation, including bone formation and fibrosis [34,63]. In most studies in adults, vitD sufficiency is associated with lower TGFβ but can be involved in more complex regulation of the cytokine in inflammatory disease [34,48,64].

IL-10, primarily produced by TH2 lymphocytes, T-regs, and monocytes, is generally referred to as an anti-inflammatory cytokine, as it inhibits the expression of many other cytokines produced during the immune response [65]. VitD sufficiency in adults has been repeatedly shown to increase IL10-mediated reduction of inflammation [65,66,67]. IL-12 promotes the TH1 phenotype and is primarily produced by macrophages and neutrophils. VitD is generally associated with lower IL-12 responses, and the IL-10/IL-12 ratio is sometimes compared in immune regulatory studies of vitD [68].

In our study, both IL-10 and IL-12 responses by stimulated infant leukocytes were higher if infants were vitD sufficient. In fact, they are the only cytokines measured that infant leukocyte antigenic responses were more closely correlated to infant’s own rather than maternal vitD status. This result with IL-10, in particular, seems to indicate that the infant’s own vitD status may be important in controlling the immune response apart from possible maternal signaling through breast milk (e.g., IL-10 downregulation of activated cytokine expression may be increased in vitD sufficient infants, which may not be discerned in this type of assay as explained below). 

Several limitations to our study left some results difficult to interpret: (a) Expectedly, the vitD status of mothers and their exclusively breastfeeding infants were closely correlated in this pilot study, making some correlations unclear. Future studies with a formula-fed group of infants should clarify those findings. Additionally, the treatment arms of the study are still blinded to the authors, so results were interpreted against measured 25-D concentration at time of blood draw (enrollment or completion). Circulating vitD metabolites and other factors (e.g., vitD binding protein), especially in maternal subjects in the study, will likely have seasonal variations, which may affect the final interpretation of trial results in association with supplementation (this will be somewhat accounted for by methods to estimate sun exposure) [15,16,50,69,70,71]. However, due to the difficulty of enrolling sufficient numbers of subjects in this study, season-specific collection or analysis of data was not possible to date. As data were analyzed with respect to circulating 25-D per se (rather than supplementation groups), some of this possible variability would likely be minimized. (b) Due to infant blood sample limitations, the same antigenic protocol was used to elicit all cytokine responses. This often resulted in extremely robust activations (e.g., IL-2) that may have obscured vitD-related differences and created excessive data deviations, requiring more subjects for statistical power. Further refinement in this approach may improve this as well. (c) As vitD-related differences in cytokine activation were measured by one method only, secretion into culture supernatant, which is most approachable through the high-throughput ELISA format, much additional information remains unclear. For example, later roles of vitamin D in the immune response, especially after activation of VDR expression in cells that may lead to anti-inflammatory effects, may be obscured in this assay. More detailed analyses of gene expression (cytokines and receptors, as well as vitD-related factors and changes to immune cell phenotypes) would likely be much more illuminating, especially since secreted protein is not likely to appreciably reduce before cultured sample collection. Such well-characterized vitD-mediated responses to immune stimulation, like T-reg proliferation, could also be elucidated [72,73,74]. (d) Spiking of some antigen-stimulated cultures with 25-D did not result in statistically significant differences in any cytokine release profile (most leukocytes can convert 25-D to 1,25-D after antigenic stimulation) [20,21]. This approach was designed to correct for outcomes possibly affected by dilution of infants’ plasma 25-D in the assay and account for infant vitD sufficiency per se (as would occur in direct supplementation), as opposed to dyad sufficiency, since dyad vitD status were often closely aligned. However, this result further indicates that some responses may have been obscured by the robust antigenic responses and method of measurement of cytokine response.

Despite these limitations, the data here clearly indicate that the vitD status of a mother can directly influence the immune response of her breastfeeding child. Thus, the roles of vitD in the development of breast milk composition result in immunological signaling to the young child [25,26,27,28,29]. Breast milk is considered largely anti-inflammatory and reduces the incidences of sepsis and necrotizing enterocolitis [75,76]. However, unlike many of the well-characterized anti-inflammatory effects of vitD sufficiency (which in most studies, are associated with the individual’s own vitD status), the apparent signal elucidated in these assays is to, at least initially, potentiate a robust antigenic response in the nascent immune system of the infant. As many aspects of vitD physiology are believed to have evolved due to pathogenic challenges [77,78,79,80,81,82,83], one could speculate that in a world of no antibiotics, no vaccines, no dietary supplementation, universal breastfeeding, and the relatively low levels of vitD metabolites generally found in breast milk to complement endogenous infant synthesis, vitD-mediated signaling through breast milk may have evolved to help protect neonates from potentially fatal infections. 

What immune-related signals associated with vitD sufficiency could a mother send to her breastfeeding baby? Epigenetic signaling associated with vitD, which begins during pregnancy, likely continues through breastfeeding [23,24,25,71,84,85,86]. Some of this signaling may involve the thousands of microRNAs found in breast milk that are thought to pass into the infant’s bloodstream and could affect the development and/or activity of the infant’s nascent immune system [84,85,86,87,88,89,90,91,92,93,94,95,96,97,98,99,100,101]. VitD has also been shown to affect the phenotypes and expression of cytokines, growth factors, immune cells, and microbiota contained in breast milk that can be transferred to the child [75,86,93,102,103].

## 5. Conclusions

During pregnancy, continued maternal vitD sufficiency in early childhood appears to play a significant role in the physiology of the breastfeeding infant, apart from the infant’s own vitD status. The ultimate goal of our future studies will be to determine the effects of maternal vitD repletion on breast milk composition and how it may improve both innate and adaptive immune development of the infant and overall health status during early childhood. 

## Figures and Tables

**Table 1:** Vitamin D status of study subjects at enrollment and completion.

Vit D Status ^1^	Mothers at Enrollment	Infants at Enrollment ^2^	Mothers at Completion ^3^	Infants at Completion ^3^
Deficiency	17	34	7	5
Insufficiency	19	21	12	12
Sufficiency	38	19	39	41
Total	74	74	58	58

^1^ Deficiency (plasma 25-hydroxyvitD ≤20 ng/mL), insufficiency (21–29 ng/mL), or sufficiency (≥30 ng/mL); ^2^ Visit 1—1 month old, exclusively breastfed infants; ^3^ Visit 4—3 months after enrollment in blinded control or treatment groups of vitD supplementation.

**Table 2:** Changes in infant plasma cytokine concentrations from study enrollment to completion.

Cytokine ^1^	Enrollment ^2^	Completion ^2^	*p*-Value ^3^	95% CI ^4^
IL-1β	0.41 ± 0.6	1.63 ± 3.5	0.004	0.41, 2.04
IL-2	0.48 ± 0.42	0.64 ± 0.68	0.14	−0.05, 0.36
IL-4	0.09 ± 0.09	0.10 ± 0.12	0.63	−0.02, 0.04
IL-6	1.09 ± 1.17	1.86 ± 2.36	0.03	0.09, 1.44
IL-8	19.3 ± 11.7	15.8 ± 6.6	0.01	−6.2, −0.81
IL-10	1.05 ± 1.22	1.33 ± 1.1	0.19	−0.14, 0.7
IL-12	0.34 ± 0.3	0.46 ± 0.35	0.04	0.01, 0.24
IL-13	1.69 ± 1.7	1.66 ± 2.4	0.94	−0.78, 0.73
IFNγ	12.1 ± 10.9	24.8 ± 29.6	0.004	4.3, 21.1
TNF	5.4 ± 1.9	6.6 ± 2.7	0.003	0.44, 1.97
TGFβ1	64,012 ± 37,882	51,741 ± 29,456	0.008	−21,132, −3410

^1^ Measured in infant plasma; ^2^ mean cytokine concentration (pg/mL) ± std. dev. at study enrollment (1 month old) or completion (4 months old); ^3^ individual enrollment to completion values compared in 58 infants by paired *t*-test (*p* < 0.05 considered significant); ^4^ confidence interval at study completion.

**Table 3:** Cytokine release after in vitro antigenic stimulation of infant whole blood.

Cytokine ^1^	Control Blood Mean Conc. ^2^ (pg/mL) ^3^	Stimulated Blood Mean Conc. ^2^ (pg/mL) ^4^	*p*-Value ^5^	Fold-Increase ^6^
IL-1β	17.4 ± 45.5	6249 ± 10174	0.0006	359
IL-2	3.1 ± 4.5	98,980 ± 104,223	<0.0001	31,929
IL-4	0.53 ± 0.67	44.4 ± 27.2	<0.0001	83.8
IL-6	247 ± 780	44,362 ± 49,062	<0.0001	180
IL-8	3720 ± 4502	83,729 ± 33,745	<0.0001	23
IL-10	3.0 ± 4.6	232 ± 180	<0.0001	77
IL-12	1.4 ± 1.7	49.5 ± 48.2	<0.0001	35
IL-13	20.3 ± 25.1	451 ± 230	<0.0001	22
IFNγ	32.0 ± 23.2	58,820 ± 43,560	<0.0001	1838
TNF	41.0 ± 88.8	20,516 ± 29,638	<0.0001	500

^1^ Measured in culture supernatants after 20 h incubation; ^2^ ±std. dev.; ^3^ 38 total V1 and V4 infant blood samples incubated without antigens; ^4^ same V1 and V4 blood samples exposed to antigen mixture; ^5^ values in control vs. stimulated blood compared by paired *t*-test (*p* < 0.05 considered significant); ^6^ mean stimulated values divided by unstimulated control.

## Data Availability

The data presented in this study are available on request from the corresponding authors.

## Supplementary Materials

The following supporting information can be downloaded at: https://www.mdpi.com/article/10.3390/nu14061266/s1, Table S1: Correlation of plasma 25-hydroxyvitamin D concentrations to cytokine release after in vitro antigenic stimulation of infant whole blood, Table S2: Vitamin D sufficiency and cytokine release after in vitro antigenic stimulation of infant whole blood, Table S3: Vitamin D sufficiency and cytokine release after in vitro antigenic stimulation of infant whole blood: Removal of statistically-calculated outliers. Table S4: Effect of 25-hydroxyvitamin D3 spike on cytokine release during in vitro antigenic stimulation of infant whole blood.
