# Supplementary material for: Maternal Vitamin D Status Correlates to Leukocyte Antigenic Responses in Breastfeeding Infants
Source: Nutrients. 2022 Mar 17;14(6):1266. doi: 10.3390/nu14061266 (PMC8952362; doi:10.3390/nu14061266)
Supplement: Supplementary file 1 [file nutrients-14-01266-s001.zip › nutrients-1623103-supplementary.pdf]

Table S1. Correlation of plasma 25-hydroxyvitamin D concentrations to cytokine release after *in vitro* antigenic stimulation of infant whole blood.

Cytokine[1]	Correlation to Maternal Plasma 25-D[2]	Correlation to Infant Plasma 25-D[2]
IL-1β	r 0.01, p 0.98	r -0.02, p 0.92
IL-2	r 0.08, p 0.63	r 0.09, p 0.61
IL-4	r 0.35, p 0.04	r 0.18, p 0.30
IL-6	r 0.16, p 0.35	r 0.26, p 0.12
IL-8	r 0.12, p 0.49	r -0.04, p 0.82
IL-10	r 0.29, p 0.08	r 0.47, p 0.004
IL-12	r 0.34, p 0.03	r 0.47, p 0.003
IL-13	r 0.26, p 0.11	r 0.13, p 0.45
IFNγ	r 0.41, p 0.08	r 0.30, p 0.23
TNF	r 0.29, p 0.07	r 0.24, p 0.15
TGFβ1	r 0.47, p 0.004	r 0.30, p 0.07

[1]Measured in culture supernatants after 20 hr incubation of antigen-stimulated infant whole blood; [2]plasma total 25-D concentration from either mother or infant linearly correlated to cytokine release from infant whole blood from same visit; Pearson's correlation coefficient is (r) value with p-value.

Table S2. Vitamin D sufficiency and cytokine release after *in vitro* antigenic stimulation of infant whole blood.

Cytokine[1]	Maternal 25-D < 30[2]	Maternal 25-D ≥ 30[2]	p-value[3]	Infant 25-D < 30[4]	Infant 25-D ≥ 30[4]	p-value[5]
IL-1β	4.2 ± 8.1	8.1 ± 11.6	0.23	6.7 ± 11.1	5.7 ± 8.7	0.80
IL-2	100.8 ± 111	97.3 ± 100	0.92	98.9 ± 109	101 ± 101	0.95
IL-4	0.034 ± 0.023	0.055 ± 0.028	0.02	0.042 ± 0.025	0.047 ± 0.034	0.64
IL-6	31.2 ± 39	56.2 ± 55	0.12	40.6 ± 44.2	54.7 ± 60	0.42
IL-8	78.7 ± 35	88.2 ± 33	0.39	84 ± 33	83 ± 38	0.93
IL-10	0.19 ± 0.15	0.27 ± 0.2	0.15	0.19 ± 0.13	0.31 ± 0.24	0.047
IL-12	0.033 ± 0.026	0.064 ± 0.059	0.049	0.040 ± 0.031	0.070 ± 0.072	0.08
IL-13	0.37 ± 0.21	0.52 ± 0.23	0.04	0.43 ± 0.21	0.48 ± 0.28	0.52
IFNγ	32.3 ± 36.3	75.7 ± 40.3	0.034	46.4 ± 36.8	68.7 ± 47.8	0.29
TNF	10.5 ± 12.9	29.5 ± 37.1	0.04	16.6 ± 23.2	29.7 ± 40.4	0.22
TGFβ1	23.0 ± 9.5	41.2 ± 29.6	0.02	27.4 ± 17.4	38.4 ± 21.9	0.12

[1]Measured in culture supernatants after 20 hr incubation of antigen-stimulated infant whole blood; [2]cytokine release (mean ng/mL ± S.D) by stimulated whole blood from infants of mothers vitD sufficient (total plasma 25-D ≥ 30 ng/mL) or insufficient (plasma 25-D < 30); [3]p-value of analyses of variance between infants grouped by maternal vitD status (calculated by unpaired t-tests or Mann-Whitney tests, as appropriate); [4]cytokine release (mean ng/mL ± S.D) by stimulated whole blood from infants grouped by their own vitD status; [5]p-value of analysis of variance between infants grouped by their own vitD status.

Table S3. Vitamin D sufficiency and cytokine release after *in vitro* antigenic stimulation of infant whole blood: Removal of statistically-calculated outliers.

Cytokine[1]	Maternal 25-D < 30[2]	Maternal 25-D ≥ 30[2]	p-value[3]	Infant 25-D < 30[4]	Infant 25-D ≥ 30[4]	p-value[5]
TNF	6.4 ± 4.9	18.6 ± 16.5	0.02	N/A[5]	N/A	N/A
TGFβ1	23.0 ± 9.5	33.8 ± 19.8	0.04	24.7 ± 11.4	35.0 ± 19.9	0.08

[1]As described for Table S2. [2,3,4]as described for Table S2, but extreme, statistically-calculated outliers removed to obtain data sets with normal distributions; t-tests re-run (as described in Methods); [5] N/A, data set did not require outlier removal (passed normality tests).

Table S4. Effect of 25-hydroxyvitamin D_3 spike on cytokine release during *in vitro* antigenic stimulation of infant whole blood.

Cytokine[1]	25-D_3 treatment (Fold-difference)[2]	p-value
IL-1β	0.99	0.95
IL-2	1.3	0.26
IL-4	0.99	0.84
IL-6	1.1	0.49
IL-8	0.91	0.33
IL-10	1.1	0.33
IL-12	0.92	0.49
IL-13	0.96	0.60
IFNγ	1.4	0.09
TNF	0.97	0.84

[1]Release measured in culture supernatants after 20 hr incubation of antigen-stimulated infant whole blood with or without addition of 50 ng/mL 25-hydroxyvitamin D_3 (TGFβ was not measured); [2]mean ratios of cytokine release with/without added 25-D.
